# EMPLOYMENT AFTER NORMAL RETIREMENT AGE: EMPLOYERS’ MOTIVES AND EXPERIENCES

**DOI:** 10.1093/geroni/igz038.472

**Published:** 2019-11-08

**Authors:** Jaap Oude Mulders

**Affiliations:** Netherlands Interdisciplinary Demographic Institute, The Hague, Netherlands

## Abstract

Due to population aging, older workers in developed countries are working much longer than previous cohorts. Some older workers even extend their careers beyond normal retirement age – or the age that is traditionally associated with retirement. While earlier work has studied employees’ motives and experiences while working after normal retirement age, motives and experiences of employers remain unexplored. Understanding employers’ perspectives is imperative for a better grasp of employees’ opportunity structures and labor market dynamics. This is especially relevant in countries with mandatory retirement systems, since here employer and employee need to negotiate a new contract after normal retirement age. I study employers’ motives to and experiences with employing older workers after normal retirement age using data from a 2017 survey among 1,312 Dutch employers. The Netherlands has mandatory retirement regulations but is also seeing an increase in employment rates after normal retirement age. Results show that 54% of employers have, in recent years, employed one or more older workers beyond their normal retirement age. This is especially common in education. 70% of employers are very positive about their previous experiences with employing older workers after normal retirement age, mostly because they had rehired older workers with unique knowledge and experience. However, employers also hardly ever took the initiative for such employment arrangements, instead leaving it to the older workers to show the desire to continue working. Although employers are largely positive, they see it as a limited phenomenon, and do not consider it a solution to labor shortages.

